# Comparison of the Relative Potential for Epigenetic and Genetic Variation To Contribute to Trait Stability

**DOI:** 10.1534/g3.118.200127

**Published:** 2018-03-21

**Authors:** Emma S.T. Aller, Lea M. Jagd, Daniel J. Kliebenstein, Meike Burow

**Affiliations:** * DynaMo Center, Copenhagen Plant Science Centre, Department of Plant and Environmental Sciences, University of Copenhagen, Copenhagen, Denmark; † Department of Plant Sciences, University of California, Davis, Davis, CA, USA

**Keywords:** Local adaptation, trait stability, DNA methylation, glucosinolates, epiQTL mapping

## Abstract

The theoretical ability of epigenetic variation to influence the heritable variation of complex traits is gaining traction in the study of adaptation. This theory posits that epigenetic marks can control adaptive phenotypes but the relative potential of epigenetic variation in comparison to genetic variation in these traits is not presently understood. To compare the potential of epigenetic and genetic variation in adaptive traits, we analyzed the influence of DNA methylation variation on the accumulation of chemical defense compounds glucosinolates from the order Brassicales. Several decades of work on glucosinolates has generated extensive knowledge about their synthesis, regulation, genetic variation and contribution to fitness establishing this pathway as a model pathway for complex adaptive traits. Using high-throughput phenotyping with a randomized block design of *ddm1* derived *Arabidopsis thaliana* epigenetic Recombinant Inbred Lines, we measured the correlation between DNA methylation variation and mean glucosinolate variation and within line stochastic variation. Using this information, we identified epigenetic Quantitative Trait Loci that contained specific Differentially Methylated Regions associated with glucosinolate traits. This showed that variation in DNA methylation correlates both with levels and variance of glucosinolates and flowering time with trait-specific loci. By conducting a meta-analysis comparing the results to different genetically variable populations, we conclude that the influence of DNA methylation variation on these adaptive traits is much lower than the corresponding impact of standing genetic variation. As such, selective pressure on these traits should mainly affect standing genetic variation to lead to adaptation.

Genetic polymorphisms in the form of *de novo* mutations and standing variation are key sources of variation in complex traits that can enable adaptation during selection. This is the foundation of the modern synthesis that linked Darwin’s theories of evolution to Mendelian Genetics (Huxley 1942). In plants, this modern synthesis is well supported by numerous studies showing the adaptive role of genetic variation controlling a range of traits including flowering time, defense metabolism and disease resistance (Wilson *et al.*, 2001; Kliebenstein *et al.*, 2001b; Bäurle and Dean 2006; Salomé *et al.*, 2011). More recently, epigenetic marks have been found to influence a number of developmental traits like flowering time and morphological traits such as root length, leaf area and internode length (Roux *et al.*, 2011; Cortijo *et al.*, 2014; Jia *et al.*, 2015; Kooke *et al.*, 2015; Rosa *et al.*, 2016). Further, some of these marks are potentially inherited across generations although the fraction of these marks that are inherited as pure epi-alleles (*i.e.*, with no associated causal genetic variation) is under intense debate (Richards 2006). These observations have led to the proposal for an “extended evolutionary synthesis” wherein heritable variation in these epigenetic marks could potentiate adaptation (Jablonka and Raz 2009; Weigel and Colot 2012; Laland *et al.*, 2014; Burggren 2016). There is a large number of studies that investigated the potential influence of epigenomic variation on a variety of traits in numerous organisms (Feinberg and Irizarry 2010; Roux *et al.*, 2011; Schmitz and Ecker 2012; Klironomos *et al.*, 2013; Olsson *et al.*, 2014; Kawakatsu *et al.*, 2016). This includes some studies that directly compared genetic and epigenetic variation, but this is a relatively small set of typically genomic studies not inherently focused on specific traits known to be under selection in a wild species (Schmitz and Ecker 2012; Olsson *et al.*, 2014; Kawakatsu *et al.*, 2016). Thus, there is a need for studies that empirically compare the relative heritability and amount of variance created by standing genetic variation *vs.* epigenetic variation for traits known to be under field selection.

One tool that has been developed to measure how epigenetic variation may influence complex traits of potential adaptive benefit is epigenetic Recombinant Inbred Lines (epiRILs) (Johannes *et al.*, 2009). In *Arabidopsis thaliana*, an epiRIL population was generated by a cross between Col-0 WT and a Col-0 *ddm1* mutant. *DDM1* encodes an ATP chromatin remodeler and its mutant shows a 30 to 70% decrease in different types of DNA methylation, CG, CHG and CHH (Johannes *et al.*, 2009; Zemach *et al.*, 2013). The progeny of this cross were backcrossed to Col-0 WT and subsequently went through several generations of selfing to develop a population of 122 lines. This generated a population where the lines contain specific and stably inherited Differentially Methylated Regions (DMRs). Thus, the epiRIL population is isogenic and the lines have distinct epigenetic marks. This allows associating phenotypic differences to DMRs within this population (Lippman *et al.*, 2004). Moreover, since DMRs are stably inherited through generations, they can be used as markers to search for epigenetic quantitative trait loci (epiQTLs). Thereby, one can both quantify and identify specific epigenomic regions correlating with trait variation.

To compare the contribution of epigenetic and genetic variation to phenotypic variation in an adaptive trait, we measured the accumulation of glucosinolate defense compounds in the epiRIL population. Glucosinolates are predominantly produced within the order Brassicales that includes many important crops such as oilseed rape and cabbage. Glucosinolates are well studied and thus, the biosynthetic pathway is often used as a model adaptive pathway based on the elaborate knowledge of enzymes and regulatory elements involved (Prasad *et al.*, 2012; Züst *et al.*, 2012; Brachi *et al.*, 2015; Kerwin *et al.*, 2015). At least 40 glucosinolates exist in *A. thaliana* (Sønderby *et al.* 2010b) with the two major glucosinolate groups being indolic glucosinolates and aliphatic glucosinolates, the latter can be further divided into short chain (SC) and long chain (LC). Glucosinolate metabolism is regulated in response to many environmental factors including herbivores and pathogens and also abiotic factors such as light and water availability (Kliebenstein *et al.*, 2005; Mewis *et al.*, 2005; Gigolashvili *et al.*, 2009; Huseby *et al.*, 2013; Mewis *et al.*, 2012; Züst *et al.*, 2012). Glucosinolate responses to attack involve complex interactions between different signaling pathways (Kliebenstein *et al.*, 2002a; Mewis *et al.*, 2005; Dam *et al.*, 2008; Frerigmann and Gigolashvili 2014; Burow *et al.*, 2015). Glucosinolates provide an adaptive benefit in the field as they aid the plant’s response and adaptation to environmental changes and thus genetics and epigenetics may have been selected for in different environments (Prasad *et al.*, 2012; Züst *et al.*, 2012; Brachi *et al.*, 2015; Kerwin *et al.*, 2015; Kerwin *et al.*, 2017).

While there is extensive information about the molecular, quantitative and evolutionary genetics of glucosinolates, there is little known about the potential for epigenetic variation to influence this pathway. We measured whether variation in DNA methylation within the epiRIL population correlates with the accumulation of different glucosinolates while simultaneously measuring adaptive flowering time trait to enable a comparison. Using a replicated randomized block design in multiple independent experiments, we quantified how variation in DNA methylation associates with the mean and the trait stability, within line variation, of these traits. This showed that epigenetic variation significantly correlates with glucosinolate accumulation and this variation differed based on the biosynthetic origins of the glucosinolates. Interestingly, there was no overlap in the epigenetic loci found to correlate with glucosinolates and the major known genes controlling natural variation in glucosinolates. Using a meta-analysis to compare the epigenetic variation in this population to literature measuring genetic variation in this trait, we showed that the influence of the epigenetic variation was dramatically lower than standing genetic variation. This was true across numerous Arabidopsis populations of different origins using both heritability and variance comparisons. Thus, selection on standing genetic variation will provide a stronger and faster response to selection than the detected epigenetic variation in the epiRILs.

## Materials and Methods

### Germplasm

122 *A. thaliana* epigenetic Recombinant Inbred Lines (epiRILs) generated in the Col-0 background and 4 Col-0 WT lines were purchased from the Versailles Arabidopsis Stock Center, Institut Jean-Pierre Bourgin. Website: http://publiclines.versailles.inra.fr/epirils/index (Johannes *et al.*, 2009).

### Experimental design

Growing of epiRILs was carried out in two independent experimental rounds. In each round, plants were grown in a randomized block design. This yielded a total of 768 plants per experiment and 1536 plants in total, leading to 12 randomized replicates of each epiRIL and 18 randomized replicates of each Col-0 WT lineage. Plants were cold-stratified for 4-6 days and subsequently grown in a light chamber for 21-22 days set to 80-120 μE/ (m^2^*s), 16 h light, 21°, 70% relative humidity. Flowering time was scored as day upon emergence of an inflorescence stem of at least 1 cm height.

### Glucosinolate extraction

Sigma-Aldrich Millipore 96 well filter plates, cat.no. MSHVN45 were charged with 45 mg DEAE Sephadex A25 and 300 µl of water per well and equilibrated at room temperature for minimum 2 hr. The water was removed using a vacuum manifold (Millipore). At day 21-23 day of growing, rosette tissue was harvested, weighted and freeze-dried before the tissue was homogenized with two stainless steel balls by shaking for 2 min at a frequency 1/30 Hz on a Mixer Mill 303 (Retsch, Haan, Germany). Glucosinolates were extracted in 300 µl 85% MeOH (v/v) containing 5 nmol p-OH-benzyl glucosinolate (extracted from seeds of Sinapis alba, SeedCom A/S, Vissenbjerg, Denmark as previously described (Thies 1979; Zrybko *et al.*, 1997) as an internal standard. Samples were centrifuged, the supernatants were applied to the filter plates and absorbed on the ion exchanger by vacuum filtration for 2-4 s. Sephadex material was washed with 2x 100 ml 70% methanol (v/v) and 2x 100 µl water and briefly centrifuged before addition of 20 µl of sulfatase solution (1.25 mg/ml, sulfatase type ^1^H, Sigma-Aldrich) per sample. After incubation at room temperature over-night, desulfo-glucosinolates were eluted with 100 µl water (Kliebenstein *et al.*, 2001b).

### Glucosinolate analysis by UHPLC/TQ-MS

1 µL of a 1:10 dilution of glucosinolates were analyzed as desulfo-glucosinolates by UHPLC/TQ-MS on an Advance-UHPLC/EVOQElite-TQ-MS instrument (Bruker) equipped with a C-18 reversed phase column (Kinetex 1.7 u XB-C18, 10 cm × 2.1 mm, 1.7 µm particle size, Phenomenex) by using a 0.05% formic acid in water (v/v) (solvent A)-0.05% formic acid in acetonitrile (v/v) (solvent B) gradient at a flow rate of 0.4 ml/min at 40°. The gradient applied was as follows: 2% B (0.5 min), 2–30% (0.7 min), 30–100% (0.8 min), 100% B (0.5 min), 100–2% B (0.1 min), and 2% B (1.4 min). Compounds were ionized by ESI with a spray voltage of +3500 V, heated probe temperature 400°, cone temperature 250°. Desulfo-glucosinolates were monitored based on the following MRM transitions: 3-methylthiopropyl (3mtp), (+)328 > 166 [5V]; 3-methylsulfinyl (3msp), (+)344 > 182 [10V]; 4-methylthiobutyl (4mtb), (+)342 > 132 [15V]; 4-methylsulfinylbutyl (4msb), (+)358 > 196 [5V]; 5-methylsulfinylpentyl (5msp), (+)372 > 210 [5V]; 7-methylthioheptyl (7mth), (+)384 > 222 [5V]; 7-methylsulfinylheptyl (7msh), (+)400 > 238 [7V]; 8-methylthiooctyl (8mto), (+)398 > 236 [5V]; 8-methylsulfinyloctyl (8mso), (+)414 > 252 [5V]; p-hydroxybenzyl (pOHB), (+)346 > 184 [10V] (internal standard). *N*- and 4-methoxy-indol-3-ylmethyl glucosinolate were distinguished based on retention times in comparison to those of known standards. Absolute quantification of the individual glucosinolates was based on response factors relative to pOHB calculated using standard curves in control extracts.

### Genetically varying populations

To compare the epiRIL variation in glucosinolates with that found in other genetic populations, we obtained foliar glucosinolate measurements from previous Arabidopsis populations that were measured at the same time under similar growth conditions using the same protocols. This minimizes the potential for technical or biological variation to dramatically influence the comparison. This included data from four previously published biparental RIL populations, Bay-0 x Sha-0 (Wentzell *et al.*, 2007), Kas x Tsu (Joseph *et al.* 2013), Ler x Cvi (Kliebenstein *et al.*, 2001a) and Ler x Col-0 (Kliebenstein *et al.*, 2002a; Kliebenstein *et al.*, 2002b). We also utilized two previously published datasets from collection of Arabidopsis accessions that was used for genome wide association mapping (Chan *et al.*, 2010; Chan *et al.*, 2011). Other populations measured for glucosinolates were not utilized because they either measured different tissues, did not measure absolute or relative glucosinolates preventing an ability to compare or did not provide the population mean data (Pfalz *et al.*, 2007).

### Statistics data visualization

All statistics were done using R version 3.3.2 (R Core Team 2016) and R studio version 0.99.491 (RStudio Team 2015). The package “car” (Fox and Weisberg 2011) and doBy was to conduct ANOVA type II to test for heritability and obtain means for the different phenotypes.

To test for heritability and obtain means for the different phenotypes, we utilized ANOVA and type II sums-of-squares based F tests using the package “car” (Fox and Weisberg 2011) and “doBy” (Højsgaard and Halekoh 2016). The model used was Phenotype = EpiRIL+Experimental_round+Tray:Experimental_round +EpiRIL:Experimental_round:Tray.

The within genotype coefficient of variation for the phenotypes was calculated separately within each of the two planting rounds, and was calculated by dividing the standard deviation for the phenotype across the individuals for each lines individuals by the mean of these same individuals. We then tested for heritability using the following model; Phenotype CV = epiRIL+Experimental_round.

### Data visualization

**Figure 1:**
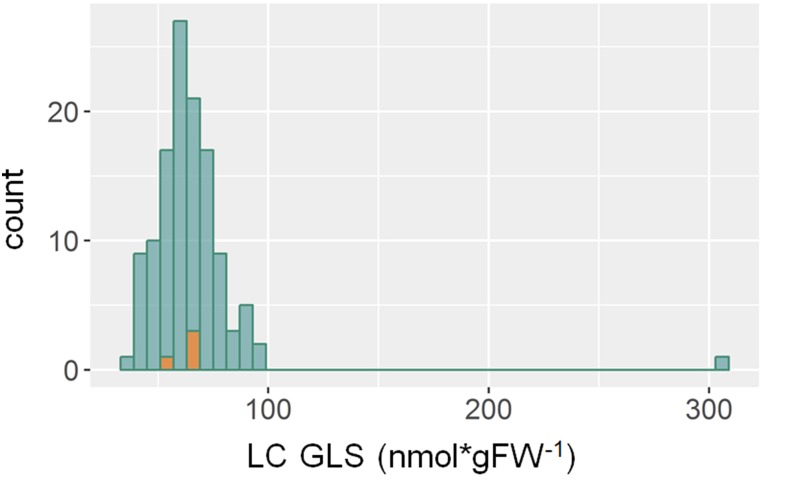
Distribution of mean LC glucosinolates among the epiRILs showing the outlier epiRIL 573. A histogram of the mean LC glucosinolate content among the 122 epiRILs is shown in green. The orange bars represent the corresponding histogram for the four WT lines.

**Figure 2:**
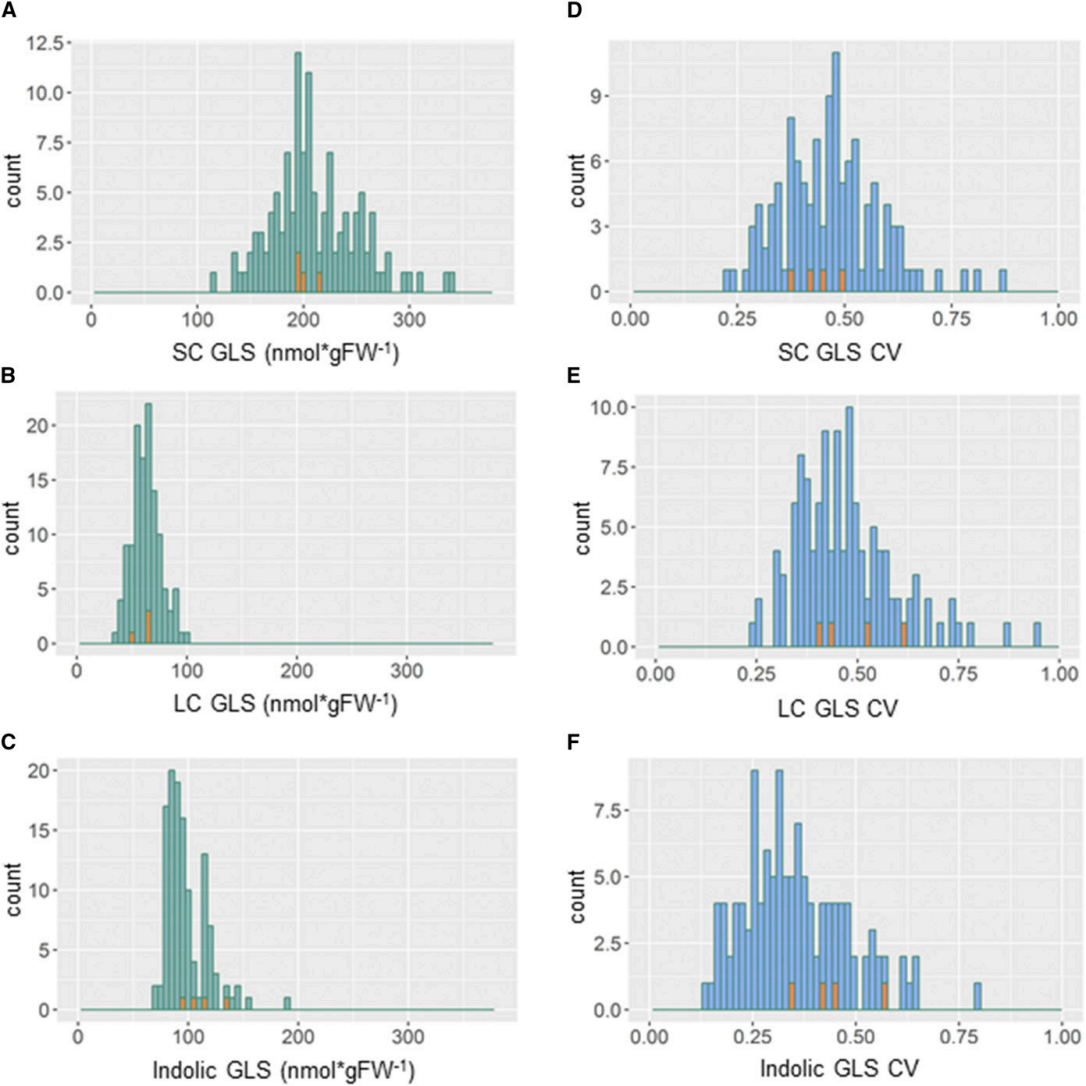
Distribution of mean and within line (CV) glucosinolate (GLS) traits among the epiRILs. Green bars show the distribution of the mean traits among the epiRILs, blue bars show the histogram of the within line variation among the epiRILs as measured by CV. Orange bars represent the distribution of the corresponding four WT lines for the specific trait shown. A) LSmean SC glucosinolate content, B) LSmean LC glucosinolate content, C) LSmean Indolic glucosinolate content, D) Within line CV for SC glucosinolates, E) Within line CV for LC glucosinolates, F) Within line CV for Indolic glucosinolates.

**Figure 3:**
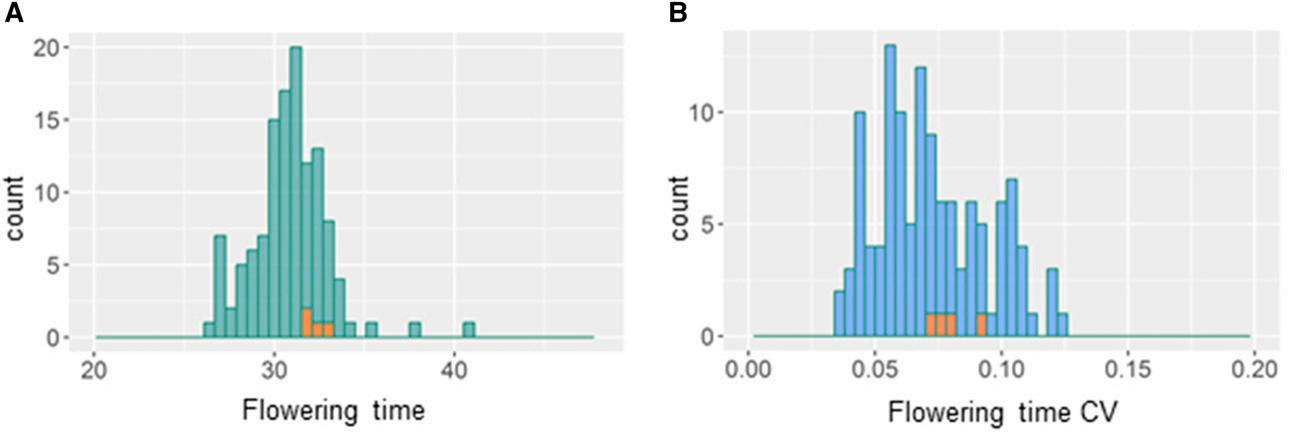
Within line distribution of flowering time mean and CV. Green bars show the distribution of the mean flowering time among the epiRILs, blue bars show the histogram of the within line variation in flowering time among the epiRILs as measured by CV. Orange bars represent the distribution of the corresponding four WT lines for the specific trait shown. A) Mean Flowering time, B) Within line CV for Flowering time.

**Figure 4:**
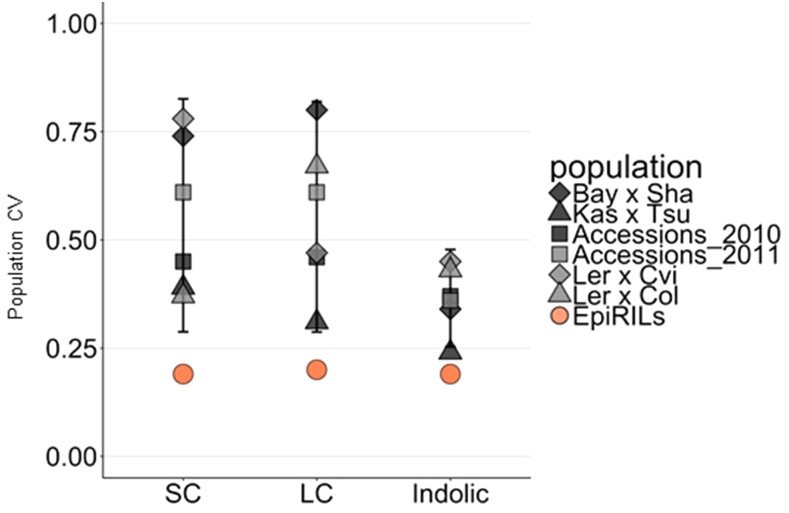
Meta-analysis of population level variation between epiRILs and genetic populations. The variation within the population (population CV) for mean glucosinolate traits is shown for epiRILs or genetic RILs or genetic association mapping populations are shown. The SC, LC and indolic traits are shown on the x-axis. Error bars for each glucosinolate mark the 99^th^ percentile confidence interval for the genetically variable populations (RILs and accessions).

Figures 1 to 4 and Figure S6 were generated in R version 3.3.2 (R Core Team 2016) and R studio version 0.99.491 (RStudio Team 2015) using the package ggplot2 (Wickham 2009). EpiQTL plots were generated from Windows QTL Cartographer Version 2.5 (Wang *et al.*, 2012).

### Composite Interval epi-QTL mapping and statistical analysis of main-effect epi-markers

EpiMarkers were obtained for the 122 epiRILs from http://publiclines.versailles.inra.fr/epirils/index (Colome-Tatche *et al.*, 2012). Windows QTL Cartographer Version 2.5 (Wang *et al.*, 2012) was used to conduct Composite Interval Mapping (CIM) (Jansen and Stam 1994; Zeng 1994). To assess significance, we conducted 1000 permutations and called significant peaks above the permutation estimated significance threshold for each trait (Churchill and Doerge 1994; Doerge and Churchill 1996). To test main effect epiMarkers and potential interactions with environment, we chose the epiMarker nearest each epiQTL peak and used this in a model tested by ANOVA in R version 3.3.2 (R Core Team 2016) using the package “car” (Fox& Weisberg, 2011 and “doBy” (Højsgaard and Halekoh 2016). Individual models were run for means and CVs on groups of SC glucosinolates, LC glucosinolates, indolic glucosinolates and flowering time. The models tested all main effect epiMarkers linked to epiQTLs for the class of phenotypes and the interaction of each marker with the experimental round.

### Data availability

File S1 contains phenotyping data for each epiRIL, *i.e.*, mean and CV for glucosinolate traits and flowering time.

## Results

### Experimental design

To test for potential epigenetic regulation of glucosinolate accumulation, we measured glucosinolate content in the *ddm1* derived isogenic epiRIL population (Johannes *et al.*, 2009). We measured glucosinolate accumulation in each line using multiple independent randomized replicates in two independent experiments. This increased the precision of the measurement on each line’s mean and produced a direct measurement of glucosinolate variation across replicates within each line as a distinct trait using the coefficient of variation (CV). We also measured flowering time in all individuals to enable a comparison with another adaptive trait previously measured in the same set of epiRILs and a collection of different genetic populations (Johannes *et al.*, 2009; Atwell *et al.*, 2010; Chiang *et al.*, 2009; Cortijo *et al.*, 2014; Kooke *et al.*, 2015).

Glucosinolate content and flowering time were measured on all 12 individual replicates of each of the 122 epiRILs and 18 individual replicates of four independent WT lines (Johannes *et al.*, 2009). All of the lines were grown in two experiments with 6 biological replicates per epiRIL per experiment and 9 biological replicates per WT line per experiment. This generated a total of 1536 plants to be analyzed for all of the phenotypes. For statistical analysis, glucosinolates were divided into specific biosynthetic groups of SC and LC aliphatic and indolic glucosinolates because these trait groups have previously been shown to have independent genetic, biosynthetic and regulatory control (Sønderby *et al.*, 2010a) (a list of all glucosinolates measured in each group is shown in Table S1).

### “Search for Outliers” Analysis of Glucosinolate and Flowering Time in EpiRILs

We first surveyed the phenotypic distributions for extreme outliers (>5 SD outliers) (Haughn *et al.*, 1991; Kliebenstein *et al.*, 2007) to test for the possibility of large effect Mendelian mutations as these may have arisen from an increased rate of transposable element transposition in the epiRILs caused by lower levels of DNA-methylation (Miura *et al.*, 2001; Kato *et al.*, 2004; Johannes *et al.*, 2009). The only phenotype that showed this level of outlier was the accumulation of LC glucosinolates where there was one epiRIL (573) with an extreme phenotype and thus potentially carrying a Mendelian mutation (Figure 1).

To test if epiRIL 573 had a potential Mendelian mutation, we back-crossed it to Col-0 WT and the F_1_ progeny was selfed to generate an F_2_ population. The F_1_ population displayed the WT phenotype and the F_2_ population showed a 3:1 segregation suggesting that the outlier LC glucosinolate phenotype in epiRIL 573 was caused by a single recessive Mendelian locus (217 plants with WT phenotype and 53 plants with epiRIL573 phenotype in F2). Because this phenotypic outlier is a unique rare outlier and potentially genetic in nature, epiRIL 573 was removed from further analysis of both glucosinolates and flowering time as a rare outlier would conflate genetic and epigenetic variance estimates in the quantitative analysis. No similar rare outliers were observed in any other phenotypes across the epiRIL population, and thus the final epiRIL population consisted of 121 epiRILs compared to 4 WT lines (Figure 2).

### Variation in DNA Methylation Significantly Correlates With Mean Glucosinolate Accumulation

Phenotypic variation in epiRILs is assumed to mainly associate with heritable variation in DMRs (Johannes *et al.*, 2009). As such, any heritability of phenotypic variation within this population is likely due to epigenetic variation. For all tested groups of glucosinolates, variation in DMRs significantly correlates with glucosinolate variation (Table 1). Linear modeling was utilized to estimate the potential epigenetic heritability (epiheritability) within this population for all measured groups of glucosinolates (Table 1). Glucosinolate variation attributable to epiheritability ranged between 10% and 13% and was thus significantly lower than that found in populations looking at natural genetic heritability of glucosinolates. In these populations, both RILs and GWAS collections, the genetic heritability ranged from ∼30–70% for indolic glucosinolates and ∼40–80% for aliphatic glucosinolates (Kliebenstein *et al.*, 2001a; Wentzell *et al.*, 2007; Chan *et al.*, 2010; Chan *et al.*, 2011; Joseph *et al.* 2013). Glucosinolate epiheritability was also lower than epiheritability for flowering time in our experiments, 32% (Table 1). This estimate of flowering time epiheritability is similar to other experiments with the exact same population suggesting that there are no major environmental or technical issues affecting our epiheritability estimates (Johannes *et al.*, 2009; Cortijo *et al.*, 2014; Kooke *et al.*, 2015). Thus, epiheritability of glucosinolate accumulation is lower than that found for flowering time and the genetic heritability found in different genetic populations.

**Table 1:** Epiheritability of mean glucosinolate content and flowering time in the EpiRILs. The Table shows the significant impact of variation between the epiRILs and experimental terms (experiment and tray) as well as interactions on measured traits using a linear model. The top of the Table shows the significance as determined from ANOVA for each term and trait epiheritability (epiRIL variance divided by total variance) is shown on the bottom row

	SC	LC	Indolic	FT
EpiRILs	0,003	0,016	<0,001	<0,001
Experiment	0,002	NS	<0,001	<0,001
Tray	<0,001	<0,001	<0,001	<0,001
ID:Experiment	NS	0,001	<0,001	<0,001
ID:Tray	NS	NS	NS	<0,001
H^2^	0,105	0,095	0,131	0,319

Using the linear model, we obtained the means for each trait in each line for further analysis. The phenotypic distribution of glucosinolate content varied between aliphatic (SC and LC) and indolic glucosinolates. SC and LC glucosinolates showed Gaussian distributions in the epiRIL population (Figure 2A, B) whereas indolic glucosinolates had a bimodal distribution (Figure 2C). SC glucosinolate phenotypes were centered around the WT lines and ranged from 113 to 341 nmol/g. LC glucosinolate content also centered around the WT lines and varied from 37 to 98 nmol/g. Indolic glucosinolate content bi-modally varied from 69 nmol/g to 187 nmol/g with two peaks at 85 nmol/g and at 115 nmol/g (Figure 2C). The four independent WT lines were dispersed across this distribution and did not correlate with the two epiRIL peaks. Flowering time varied across the epiRILs from 26 days to 41 days and had a Gaussian distribution (Figure 3A). In contrast to aliphatic glucosinolates, the distribution of flowering times among epiRILs did not center around the WT sample lines, but instead showed a high skew toward earlier flowering time with only a few epiRILs flowering later than WT. This suggests that variation in DMRs within this background can equally decrease and increase glucosinolate content but shows a bias toward earlier flowering time. As such, there is a bias in the directionality of how *ddm1*-mediated methylation changes associates with the glucosinolate and flowering time phenotypes.

### Correlation Between DNA Methylation variation and Within Line Variation in Glucosinolate Accumulation

We also tested how DMR variation correlates with phenotypic stability within a line using the within line coefficient of variation (CV). When looking at line mean only, we are blind to the dispersion of the replicates of a line, which could potentially play an important role in adaption (Hall *et al.*, 2007; Ordas *et al.* 2008). The experimental design allowed us to obtain independent measures of the within line CV per epiRIL and directly test if DMR variation correlates with this dispersion. While epiheritability of within line CV was not statistically significant for SC and indolic glucosinolates or flowering time, there was a suggestive difference for LC glucosinolates (Table 2). Interestingly, the estimated epiheritability of within line CV was higher than that found for the mean. For glucosinolates it ranged between 36% and 51% and flowering time showed 41% heritability for trait CV. This estimate of within line CV epiheritability is very similar to that found for genetic heritability of within line CV in *Arabidopsis for* the phenotype (Joseph *et al.* 2014). The lower statistical significance for the within line CV epiheritability in comparison to the mean is likely because there were only two independent measurements, one per experiment, for this trait in comparison to 12 independent measurements for the mean (Table 1).

**Table 2:** Epiheritability of within line variation as measured by CV for glucosinolate content and flowering time. The Table shows the significant impact of variation between the epiRILs and experiments on measured traits using a linear model. The top of the Table shows the significance as determined from ANOVA for each term and trait epiheritability (epiRIL variance divided by total variance) is shown on the bottom row

	SC	LC	Indolic	FT
EpiRILs	NS	0,064	NS	NS
Experiment	<0,001	<0,001	<0,001	<0,001
H^2^	0,474	0,507	0,360	0,406

Using the mean for the within line CV for all the epiRILs showed that the trait distributions for all glucosinolate groups appeared similar (Figure 2D, E, F). All were Gaussian distributed, ranged between 0,2 and 1 and had a peak around 0,5. WT lines were in the peak of epiRILs for SC- and LC glucosinolates and appeared slightly skewed toward higher CV for indolic glucosinolates. Thus, while the trait distributions for the mean accumulation of the different glucosinolates differed in epiRILs, the within line CV was very similar across all of the glucosinolates (Figure 2A-E). Within line CV for flowering time was overall lower than for glucosinolates (Figure 3B). CV values ranged between 0,03 and 0,4 and showed a more dispersed distribution than aliphatic glucosinolates (SC and LC glucosinolates). Thus, within line CV for different traits appears to be associated with DMRs within this population. Moreover, also for CV, flowering time and glucosinolates are distinct traits from each other and the *ddm1* epi-QTL are not pleiotropically altering the entire plant.

### Comparing Genetic and Epigenetic Influence on Glucosinolate Population Variation

Previous publications using the same experimental design to measure genetic variation in glucosinolates allowed for a direct comparison with the DMR variation (Kliebenstein *et al.*, 2001a; Kliebenstein *et al.*, 2002b; Wentzell *et al.*, 2007; Chan *et al.*, 2010; Chan *et al.*, 2011; Joseph *et al.* 2013). In combination with the previous comparison of heritability to epiheritability, this can give insight to whether the epigenetic influence on phenotypic variation was similar or different from genetic influence. To facilitate this meta-comparison, we calculated the population CV (an estimate of the range of phenotypic variance) for each trait across the epiRIL and genetic populations using the mean trait values. Using the population CV, we compared the epiRIL population trait variation to the trait variation in four RIL populations based on crosses of Bay x Sha, Kas X Tsu, Ler x Cvi and Ler x Col (Kliebenstein *et al.*, 2001a; Kliebenstein *et al.*, 2002a; Kliebenstein *et al.*, 2002b; Wentzell *et al.*, 2007; Joseph *et al.* 2013). In addition to the RIL populations, we compared the epiRIL population to two studies on *A. thaliana* accessions (Chan *et al.*, 2010; Chan *et al.*, 2011) (for details on populations, see materials and methods). For all groups of glucosinolates, the epiRILs had the lowest population CV (around 20%), which was below the 99 percentile confidence interval calculated for genetic variation using the different RIL and accession populations (Figure 4). This shows that the DMR variation caused by the *ddm1* mutation within the epiRIL variation associates only with a small fraction of the variation possible from natural genetic variation.

Besides being lower than the other populations, the epiRIL population was also the only population showing the same level of population CV for all glucosinolate groups. The genetically differing populations had varying population CVs for the different glucosinolate biosynthetic groups with overall lower indolic glucosinolate population CV. Similarly, the flowering time population CV in the epiRIL population was dramatically lower than that found in a collection of accessions, 7% epigenetic population CV and 62% for accessions (Atwell *et al.*, 2010). This points to the potential epigenetic contribution to phenotypic variation caused by the DMR variation being much lower than that found for genetic variation for both glucosinolates and flowering time.

### Mapping EpiQTLs for Glucosinolate Levels

**Figure 5:**
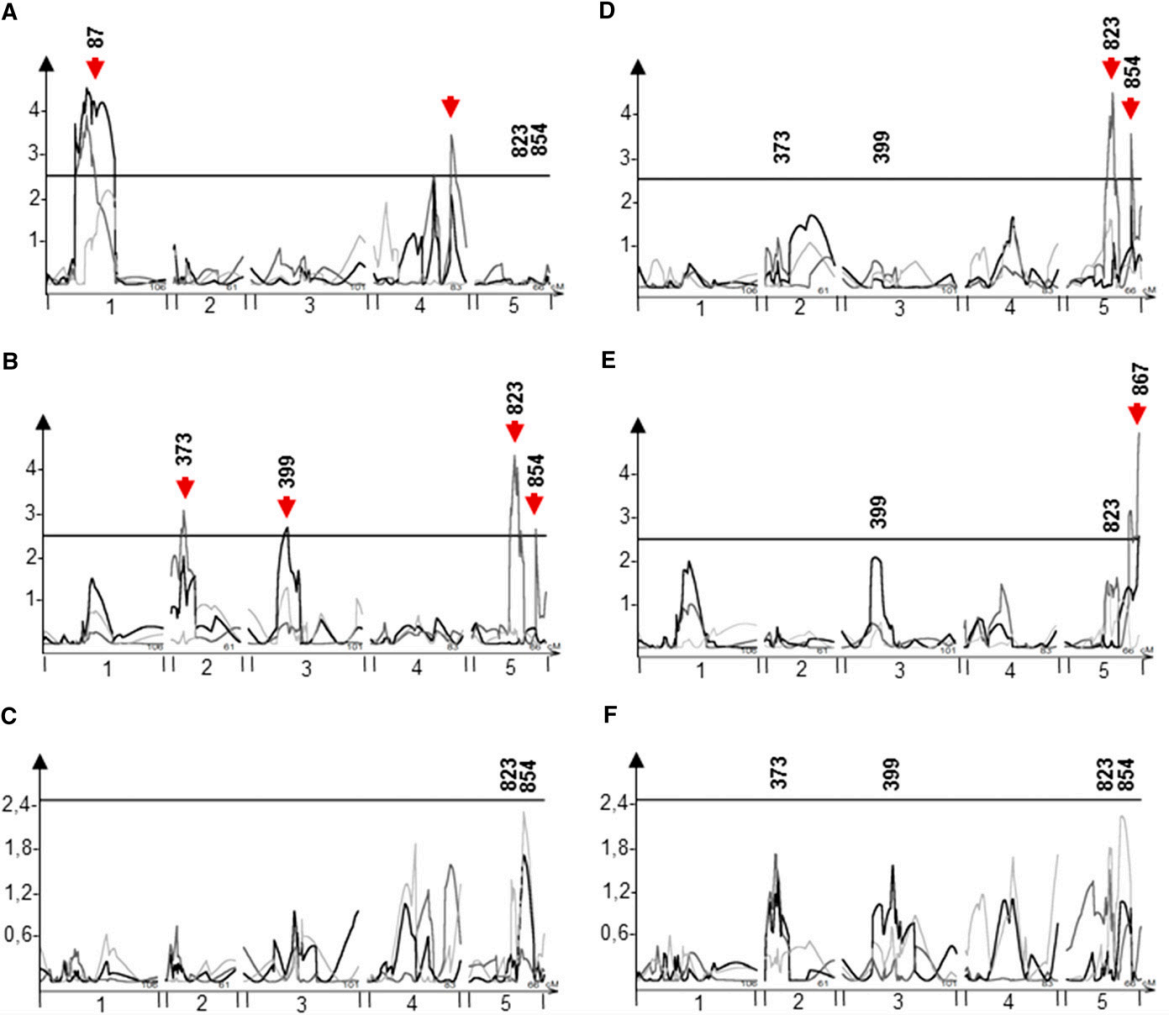
EpiQTL mapping of SC glucosinolate means. Plots show composite interval mapping results and significance estimated from 1000 permutations. The x-axis shows the genome by chromosome and the y-axis shows the LOD score. The significance thresholds are plotted for each trait and significant QTL are re marked with red arrows. The light gray line shows the QTL map using the means from experiment 1, dark gray shows experiment 2, and black lines represent the pooled data from experiment 1 and 2. Marker names show the position of significant makers after using a linear model to assess loci. EpiQTLs not assigned a marker were rejected in the subsequent ANOVA. Glucosinolate abbreviations are shown in Table S1. Analyzed SC glucosinolates are A) 3MTP, B) 3MSP, C) 4MTB, D) 4MSB, E) 5MSP, F) Pooled SC glucosinolates.

To identify DMRs linked to the observed variation of glucosinolate accumulation, we mapped epigenetic quantitative trait loci (epiQTLs) in the epiRIL population. We used the available map of 126 DMRs for the 121 epiRILs to locate epiQTLs (Colome-Tatche *et al.*, 2012) and performed composite interval mapping (CIM) per experiment and across experiments with 1000 permutations to test for significance. All individual glucosinolates and the pooled data for each biosynthetic group were used to map epiQTLs. As shown for SC glucosinolates, epiQTLs were found for 3MTP, 3MSP, 4MSB and 5MSP glucosinolate accumulation (Figure 5A, B, D, E). Some epiQTLs had broad affects across a set of metabolites such as one epiQTL on chromosome 5 that showed up for 3MSP, 4MSB and 5MSP glucosinolates (Figure 5B, D, E). In contrast, other epiQTLs were specific for a single glucosinolate as in the case of epiQTLs on chromosome 2 and 3 that only showed up for 3MSP (Figure 5B). After locating epiQTLs, we used a linear modeling approach involving all identified epiQTLs to identify the marker with the lowest P-value for each epiQTL. We included the total list of candidate epiQTLs identified for all individual SC glucosinolates and pooled SC glucosinolates within the linear model. This enabled the identification of significant loci not identified using the CIM approach, such as identified 3MSP epiQTL on chromosome 3 centered on marker 399 (Figure 5B); with the modeling approach, this locus was also found to alter the accumulation of 4MSB, 5MSP and pooled SC glucosinolates suggesting that it is a locus altering the accumulation of all sulfinyl SC glucosinolates (Figure 5B, D, E, F). In contrast, an epiQTL on chromosome 5 centered on marker 823 influenced variation of all SC glucosinolates, both methylsulfinyl and methylthiol. In summary, markers were found that linked to all SC glucosinolates, while others were specific for either one SC glucosinolate or subgroups of SC glucosinolates as in the case of methylsulfinyls. LC glucosinolates showed similar patterns, as marker 1 was specific to methylthiols, whereas marker 373 influenced all individual glucosinolates (Figure S1). Indolic glucosinolates showed a slight different pattern when looking at the individual glucosinolates in the biosynthetic group (Figure S2). Most markers were specific to one indolic glucosinolate, whereas marker 859 was significant for all except NMOI3M. NMOI3M did show an epiQTL in this region, but this epiQTL was rejected in the subsequent modeling. Thus, we identified epiQTLs that associated either with an array of glucosinolate accumulation or specific subsets. This blend of specific and general epiQTLs is similar to observations found for QTL analysis of natural genetic variation (Kliebenstein *et al.*, 2001a; Wentzell *et al.*, 2007; Joseph *et al.* 2014).

**Figure 6:**
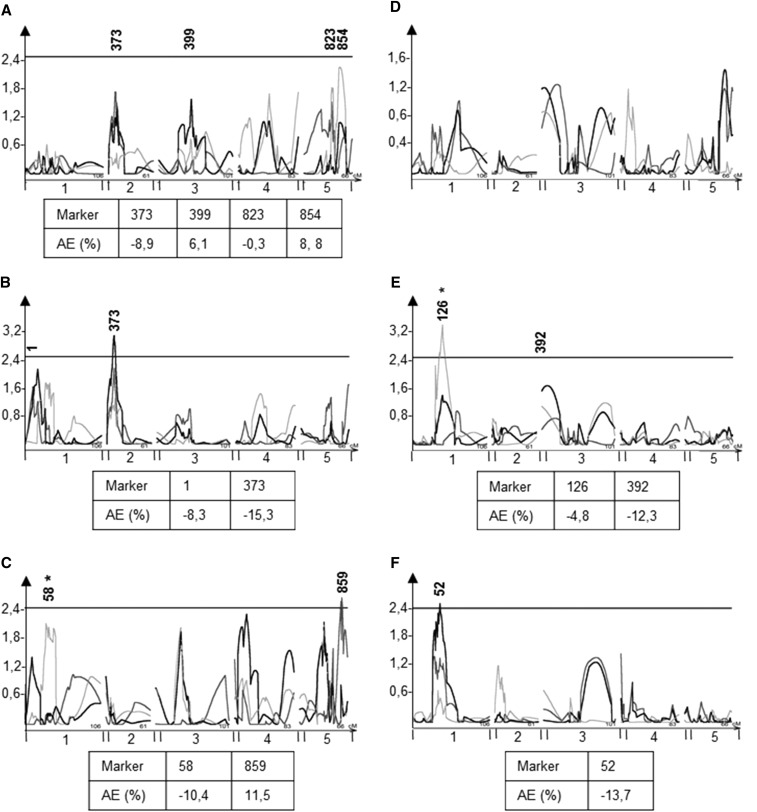
EpiQTL mapping of glucosinolate mean and CV. Plots show composite interval mapping results and significance estimated from 1000 permutations. The x-axis shows the genome by chromosome and y-axis shows the LOD score. The significance thresholds are plotted for each trait and significant QTL are re marked with red arrows. The light gray line shows the QTL map using the means from experiment 1, dark gray shows experiment 2, and black lines represent the pooled data from experiment 1 and 2. Marker names show the position of significant makers after using a linear model to assess loci. EpiQTLs not assigned a marker were rejected in the subsequent ANOVA. An asterisk after a marker shows that the marker was not significant in both experiments. Beneath plots are shown the additive effect of markers, *i.e.*, the percentage phenotypic change when the marker is *ddm1* within the epiRIL lines compared to WT. A) Mean SC glucosinolate content, B) Mean LC glucosinolate content, C) Mean Indolic glucosinolate content, D) Within line CV for SC glucosinolates, E) Within line CV for LC glucosinolates, F) Within line CV for Indolic glucosinolates.

To calculate the effect size of these found epiQTLs, we estimated the additive effect as the percentage difference in phenotypic means of plants when the methylation status of the epiQTL was as the *ddm1* parent compared to same marker originating from WT (Figure 6). For SC glucosinolates, marker 399 and 854 correlated with increased glucosinolate accumulation when originating from *ddm1* compared to WT, while marker 373 and 823 correlated with decreased glucosinolate accumulation when originating from *ddm1* (Figure 6A). Marker 373 also correlated with decreased LC glucosinolate accumulation albeit with a larger effect for LC glucosinolates (Figure 6A, B). Markers found for indolic glucosinolate means also correlated with both increases and decreases in additive effect (Figure 6C), as the two markers, 58 and 859, had a respective a 10,4% decrease and 11,5% increase of the *ddm1* derived allele compared to WT (Figure 6C). Thus, the *ddm1* derived epialleles associated with a mixture of positive and negative effects on aliphatic glucosinolate accumulation further showing that DDM1-controlled methylation variation can correlate with both increase and decrease in glucosinolate accumulation.

### Flowering time epiQTLs

**Figure 7:**
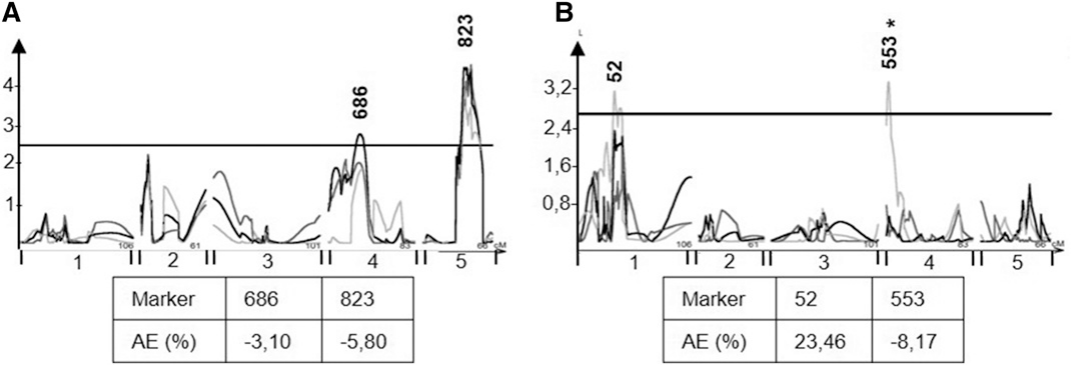
EpiQTL mapping of flowering time mean and CV. Plots show composite interval mapping results and significance estimated from 1000 permutations. The x-axis shows the genome by chromosome and y-axis shows the LOD score. The significance thresholds are plotted for each trait and significant QTL are re marked with red arrows. The light gray line shows the QTL map using the means from experiment 1, dark gray shows experiment 2, and black lines represent the pooled data from experiment 1 and 2. Marker names show the position of significant makers after using a linear model to assess loci. An asterisk after a marker shows that the marker was not significant in both experiments. Beneath plots are shown the additive effect of markers, *i.e.*, the percentage phenotypic change when the marker is *ddm1* within the epiRIL lines compared to WT. EpiQTLs not assigned a marker were rejected in the subsequent ANOVA. A) Mean Flowering time, B) Within line CV for Flowering time.

As a control to compare the epiRIL population with previous publications, we mapped epiQTLs associated with flowering time as described above (Figure 7). This analysis identified two epiQTLs for flowering time that centered on marker 686 on chromosome 4 and marker 823 on chromosome 5 which are the same makers as previously identified to correlate with flowering time (Figure 7A) (Cortijo *et al.*, 2014, Kooke *et al.*, 2015). The additive effect on flowering time was a 3,1% decrease for the *ddm1* derived allele at marker 686 and 5,8% decrease for the *ddm1* derived allele at marker 823. Compared to previous studies (Cortijo *et al.*, 2014; Kooke *et al.*, 2015), both markers showed less correlation with the phenotype in our experiment. The fact that we identified the same flowering time loci as previously found suggests that this population is behaving as in previous studies allowing us to infer that any differences between glucosinolates and flowering time effects in this population are not the result of differences between laboratories and experimental designs. Further, this shows again that DDM1-mediated methylation variation correlates with earlier flowering in this population.

### EpiQTLs Identified for Within Line Trait Variance

The replicated experimental design allowed for the direct measurement of variation between replicates of the same epigenotype as a phenotype that can be mapped to assess if variation in epigenetic marks associates with the stochastic variation or robustness of specific lines. Using phenotypic CV within each line as the measure of variation between replicates, we found epiQTLs that link to within line dispersion for all of the measured traits except SC glucosinolates (Figure 6D, F, Figure S3, Figure S4, Figure S5). For LC glucosinolates, we identified marker 126 on chromosome 1 and marker 392 on chromosome 3 (Figure 6E). Both of these loci have additive effects whereby the *ddm1* derived allele links to lower within line variance. Critically, neither of these LC glucosinolate CV markers correlated with the average accumulation of LC glucosinolates within the epiRIL population suggesting that as with genetic QTL, epiQTLs can be specific for either mean glucosinolate accumulation or within line variation showing that these are as affected as separate traits by DDM1-mediated epigenetic marks (Figure 6B, E). In contrast to LC glucosinolate CV, the only identified CV epiQTL for indolic glucosinolates, marker 52 on chromosome 1 was in a region also correlating with indolic glucosinolate mean accumulation (Figure 6C, F). For indolic glucosinolate means however, it was the neighboring marker 58 explaining most of the epiQTL. The two markers are situated ∼200 kbp away from each other indicating that they may be different loci.

Similar to LC glucosinolate CV, two epiQTLs were identified for flowering time CV and these did not link to mean flowering time (Figure 7A, B). These epiQTLs for flowering time CV have not been previously identified (Kooke *et al.*, 2015). Taken together, it is possible to find epiQTLs that associate both within line (CV) and between line variation of glucosinolate accumulation and flowering time. Further, this indicates that DDM1-mediated DNA methylation can influence phenotypic stability within individual lines.

### EpiQTLs are Largely in Genomic Regions Unknown for Glucosinolate Phenotypes

**Figure 8:**
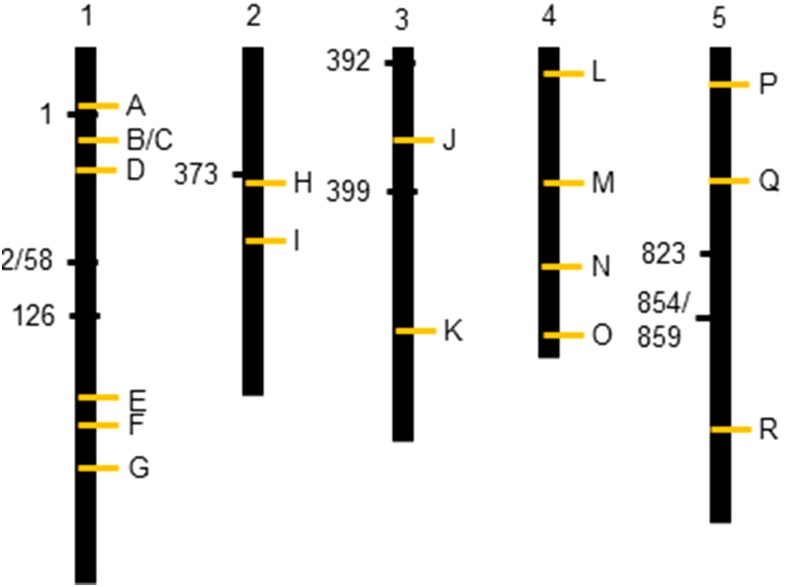
Genomic position of significant epiQTL markers and glucosinolate genes. The markers associated with significant epiQTL are shown to the left of each chromsome. Letters denote glucosinolate genes: A, AT1G12140 *FMO GS-OX5*. B, AT1G16410 *CYP79F1* and AT1G16400 *CYP79F2*. C, AT1G18590 *SOT17*. D, AT1G24100 *UGT74B1*. E, AT1G62540 *FMO GS-OX2* and AT1G62560 *FMO GS-OS3* and AT1G62570 *FMO GS-OX4*. F, AT1G65860 *FMO GS-OX1*. G, AT1G74100 *SOT16* and AT1G74090 *SOT18*. H, AT2G20610 *SUR1*. I, AT2G31790 *UGT74C1*. J, AT3G19710 *BCAT4*. K, AT3G49680 *BCAT3*. L, AT4G03060 *AOP2* and AT4G03050 *AOP3*. M, AT4G13770 *CYP83A1*. N: AT4G30530 *GGP1*. O, AT4G39950 *CYP79B2* and AT2G22330 *CYP79B3*. P, AT5G07690 *MYB29* and AT5G07700 *MYB76*. Q, AT5G23010 *MAM1* and AT5G23020 *MAM3*. R, AT5G61420 *MYB28*.

To test if any of the identified epiQTLs may co-locate with known glucosinolate genes, we compared the position of significant epiQTLs with a large collection of known glucosinolate biosynthetic and major regulatory genes (Figure 8). The vast majority of epiQTLs had no overlap with known glucosinolate genes suggesting that they are linked previously unknown causal loci (Figure 8, see marker 52, 58, 126, 392, 823 and 854). Three markers, 373, 399 and 859, were in proximity to glucosinolate loci but the marker closest to the glucosinolate gene was not having the highest LOD score, suggesting DNA methylation of other genetic regions correlating with the phenotype. Marker 1 was the only marker that appeared to link to a known glucosinolate gene as it is located between the *CYP79F1/2* and *FMO GS-OX5* enzymatic genes. Marker 1 is located >1.2 Mbp away from the *CYP79* locus, which is too far to indicate a link between the two. However, it is approximately 200 kbp away from *FMO GS-OX5*. *FMO GS-OX5* is involved in LC glucosinolate synthesis and marker 1 was identified for LC glucosinolates. Together, this might indicate that the methylation status of *FMO GS-OX5* is linked to the LC glucosinolate variation observed in epiRILs. To test whether marker 1 associates with this gene, we plotted the additive effect of marker 1 on the ratio between methylthio glucosinolates (7MTH and 8MTO) and total LC glucosinolates which represents a direct approximation of this enzymes function (Figure S6) (Li *et al.*, 2008). This did not point to a correlation between *GS-OX5* and marker 1 as the difference in tested ratio levels did not show a significant difference when marker 1 originated from *ddm1* compared to WT. Thus, the epiQTLs found are large epigenomic blocks and thus can span many genes. This makes it impossible to precisely define what is correlating to the traits without doing a fine-scale mapping. We can however, conclude that the identified epiQTLs do not appear to link to known glucosinolate genes suggesting that the *ddm1* derived epiQTLs are linked to a different suite of genes. This is in contrast to QTL for natural genetic variation of glucosinolates wherein a large number of the loci are due to causal polymorphisms in the biosynthetic enzymes and key regulatory genes (Kliebenstein *et al.*, 2001a; Wentzell *et al.*, 2007).

## Discussion

Recent work has implied that epigenetic marks may play a role in the inheritance and evolution of adaptive traits (Roux *et al.*, 2011; Cortijo *et al.*, 2014; Jia *et al.*, 2015; Kooke *et al.*, 2015; Rosa *et al.*, 2016). In this study, we utilized an epiRIL population that varies in *DDM1* ediated DNA methylation to test for the potential to influence the heritability of a set of adaptive traits in *Arabidopsis thaliana*; glucosinolate defense compounds and flowering time (Johannes *et al.*, 2009). We show that both glucosinolates and flowering time display significant epiheritability, meaning that that DMRs associate with the heritable variation of these adaptive traits (Table 1, Table 2). Moreover, there were specific epiQTLs that were unique for each adaptive trait showing that the epiheritability can alter specific subsets of adaptive traits and that they are not globally pleiotropic. Similar to genetic variation, it was possible to find epigenomic regions correlating with the variation of both mean variation between lines and stochastic variation within lines (CV) and some of these regions were specific for controlling the within line variance (Figure 6). As such, epigenetic variation has the potential to influence the inheritance of these adaptive traits.

Existing literature that measured the standing genetic variation affecting these same adaptive traits in *Arabidopsis thaliana* using the same experimental design and conditions provided the ability to directly compare genetic variation and DDM1-mediated epigenetic variation (Kliebenstein *et al.*, 2001a; Kliebenstein *et al.*, 2002b; Wentzell *et al.*, 2007; Chan *et al.*, 2010; Chan *et al.*, 2011; Joseph *et al.* 2013). Genetic heritability in both RIL populations and accession collections was dramatically higher than that found in the epiRIL population. Further, the epiRIL population had a significantly lower range of phenotypic variation than the genetic populations (Figure 4); the epigenetic variation in the epiRIL population was ranging between 1,2 to 4 fold lower than the standing genetic variation for the same trait. Thus, if the epiRIL population provides an accurate representation of the potential for epigenetics to influence adaptive trait variation, the standing genetic variation provides a vastly larger pool of phenotypic diversity that is also of higher heritability. As such, selection on this standing genetic variation will provide both a stronger and faster response to selection than the epigenetic variation in the epiRILs. Proving this hypothesis however requires future studies in other epiRIL populations that vary either for different individual epigenetic marks or for a blend of epigenetic marks simultaneously. The large level of standing genetic variation for these traits argues that parsing out the more subtle epigenetic influences in existing genetic populations will be complicated.

### Phenotypic Variation in EpiRILs and Potential Mechanistic Insight

The distribution of aliphatic glucosinolates in the epiRILs (Figure 2A, B) showed a Gaussian distribution centered around the WT values. This shows that DMRs link to both positive and negative changes in the accumulation of aliphatic glucosinolates. In contrast, flowering time distributions of epiRILs showed a skew toward earlier flowering compared to WTs (Figure 3A). This might be the overall reduction in DNA methylation levels in the epiRIL that correlates with this earlier onset of flowering time. This suggests that there is a fundamental difference in how DDM1 methylation is linking to these adaptive traits. The observation that DMR variation in epiRILs only associates with earlier flowering times suggests that this methylation may contribute to the irreversible switch like behavior of flowering, which cannot reverse when having started the process of flowering. Previous studies also showed that epiRILs flowered earlier than the parental Col-0 controls grown along when making the epiRILs (Johannes *et al.*, 2009, Table S3). However, the *ddm1* mutant parent has been shown to flower either earlier or later than WT parent making the direction of the flowering phenotype based on *ddm1*-caused hypo-methylation unclear (Soppe *et al.*, 2000; Johannes *et al.*, 2009; Roux *et al.*, 2011). In contrast, glucosinolates are not regulated as an irreversible switch and can be repressed after being induced or vice versa. Thus, DDM1-mediated methylation links to both the activation and repression of glucosinolates to aid in the proper adjustment of glucosinolate levels.

### Lack of Overlap in EpiQTLs With Biosynthetic Genes

An interesting observation in this study is that we did not identify instances where the glucosinolate biosynthetic enzyme genes were within an epiQTL region suggesting that the epiQTLs largely do not influence the biosynthetic genes. This is somewhat in contrast to QTL mapping studies in Arabidopsis RILs, which showed that the large-effect variants linked to glucosinolate accumulation are almost entirely in biosynthetic loci (Kliebenstein *et al.*, 2001a; Lambrix *et al.*, 2001; Textor *et al.*, 2004; Kroymann and Mitchell-Olds 2005; Hansen *et al.*, 2007; Sønderby *et al.*, 2007; Hansen *et al.*, 2008; Sønderby *et al.*, 2010a). In the natural accessions, this is similarly true that the major effect polymorphism are in biosynthetic loci (Chan *et al.*, 2010; Chan *et al.*, 2011; Brachi *et al.*, 2015). However, there is also a vast universe of loci that appear to have causal polymorphisms each with small effects (Chan *et al.*, 2010; Chan *et al.*, 2011). As such, any enrichment of genetic causation is only identified within the large-effect polymorphisms. Interestingly, these large effect loci are associated with different epigenetic marks but this is a side-effect of the genetic inversions and duplications present in these genes (Kroymann *et al.*, 2003; Chan *et al.*, 2010; Schmitz and Ecker 2012; Schmitz *et al.*, 2013; Kawakatsu *et al.*, 2016). Thus, it is possible that DDM1 methylation only has the ability to causally influence the peripheral small effect loci within this epiRIL population. Future work studying other epigenetic marks will be required to come up with a reason for this difference.

### EpiQTL Mapping of Within *Vs*. Between Line Variation

Dispersion (or stochastic variation) between replicates of homozygous lines is a trait that is under genetic control and is potentially adaptive (Queitsch *et al.* 2002; Ansel *et al.*, 2008; Raj and van Oudenaarden 2008; Sangster *et al.* 2008; Veening *et al.*, 2008; Jimenez-Gomez *et al.*, 2011; Joseph *et al.*, 2015; Lachowiec *et al.*, 2016). These loci can be specific to stochastic variation within lines or can also associate with the mean variation between independent genotypes (Lachowiec *et al.*, 2016). Given the potential for epigenetics to create this stochastic within line variation, we tested the possibility for the epiRILs to have different within line dispersion/stochastic variation within otherwise homozygous epigenotypes. Using within line CV to directly map variation in dispersion, we identified epiQTLs that associated with variation in within line CV for all of the traits from glucosinolate accumulation to flowering time. Previous efforts to identify epiQTLs that linked to within line CV for flowering times did not find these loci (Kooke *et al.* 2015). The most likely explanation for this difference is that in our experiments, we had independent replication across experiments on within line CV allowing for more power to identify these loci. Thus, the methylation status of the specific epiQTLs specifically associate with within line dispersion and not only the means of glucosinolates and flowering time. Interestingly, despite the mean distributions differing between aliphatic and indolic glucosinolates, their CV distributions were very similar (Figure 2 D, E, F). This is in contrast to flowering time that showed very low variation of within line CV (Figure 3B). Like genetic QTL for within line CV, these epiQTLs were a blend of loci that specifically influence within line CV and loci that shift both within line and between line variation. Thus DDM1-mediated methylation associates with phenotypic stability within a line similar to genetic polymorphisms.

In summary, this shows that while epigenetic mark variation correlates with glucosinolate variation, it is at a point that is much lower than the standing genetic variation. Thus, variation in DDM1-mediated epigenetic marks is unlikely to have a predominant if any influence on adaptation in a polymorphic population via flowering time or glucosinolates. This suggests that any role of epigenetic variation in influencing adaptation is most likely to be identified in isolated homozygous populations that have little to no migration with neighboring populations. Future work is required to assess if variation in other epigenetic marks may have more potential influence on adaptive traits.

## Supplementary Material

Supplemental Material is available online at www.g3journal.org/lookup/suppl/doi:10.1534/g3.118.200127/-/DC1.
