# Supplementary material for: Comparison of the Relative Potential for Epigenetic and Genetic Variation To Contribute to Trait Stability
Source: G3 (Bethesda). 2018 Mar 21;8(5):1733–46. doi: 10.1534/g3.118.200127 (PMC5940164; doi:10.1534/g3.118.200127)
Supplement: Supplementary file 3 [file 1733FigureS3.pdf]

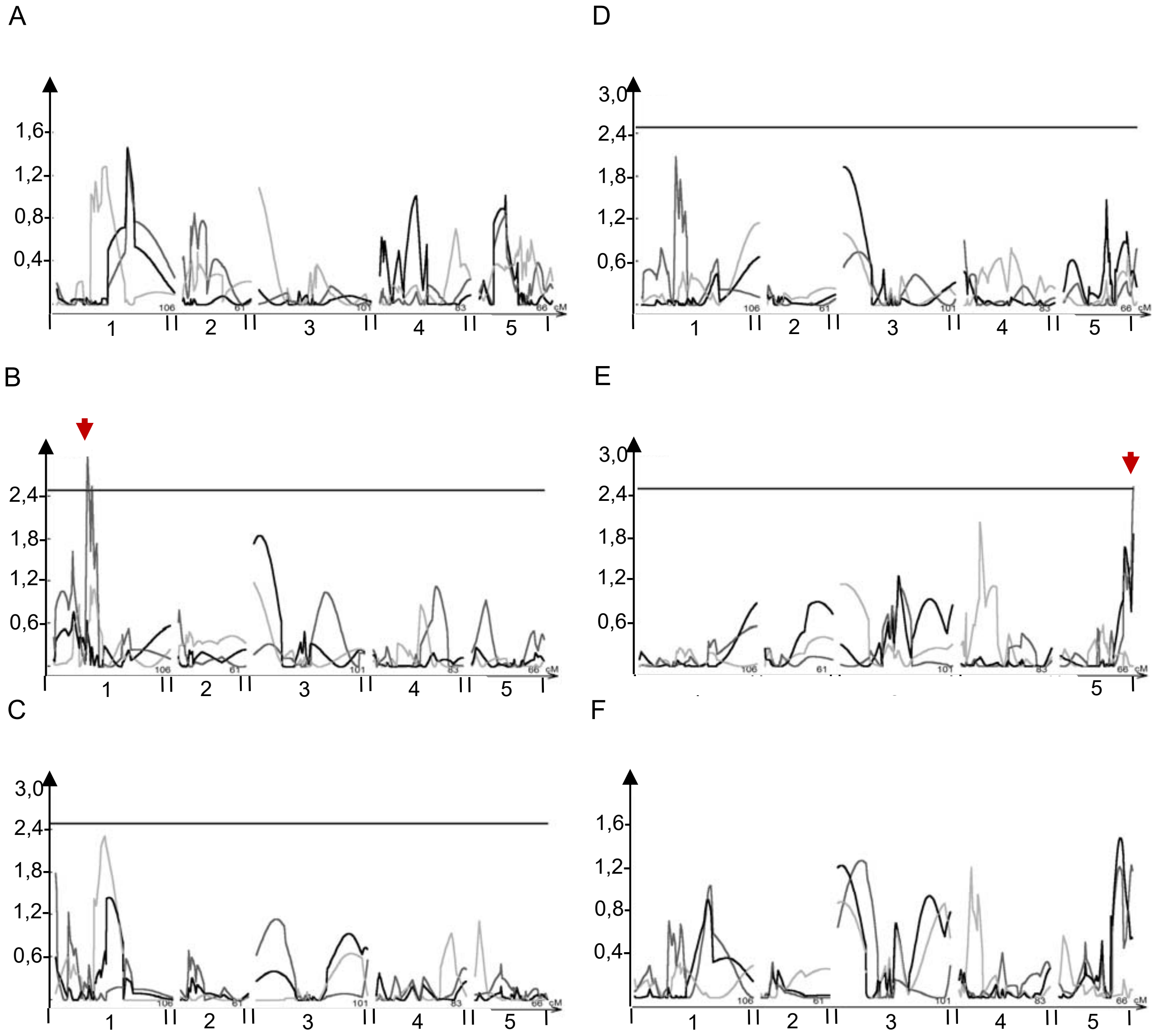

Figure S3: EpiQTL mapping of SC glucosinolate CV. Plots show composite interval mapping results and significance estimated from 1000 permutations. The x-axis shows the genome by chromosome and y-axis shows the LOD score. The significance thresholds are plotted for each trait and significant QTLs are re marked with red arrows. The light grey line shows the QTL map using the means from experiment 1, dark grey shows experiment 2, and black lines represent the pooled data from experiment 1 and 2. Marker names show the position of significant makers after using a linear model to assess loci. An asterisk after a marker shows that the marker was not significant in both experiments. EpiQTLs not assigned a marker were rejected in the subsequent ANOVA. Analyzed SC glucosinolates are A) 3MTP, B) 3MSP, C) 4MTB, D) 4MSB, E) 5MSP, F) Pooled SC glucosinolates.
