# Supplementary material for: Comparison of the Relative Potential for Epigenetic and Genetic Variation To Contribute to Trait Stability
Source: G3 (Bethesda). 2018 Mar 21;8(5):1733–46. doi: 10.1534/g3.118.200127 (PMC5940164; doi:10.1534/g3.118.200127)
Supplement: Supplementary file 6 [file 1733FigureS6.pdf]

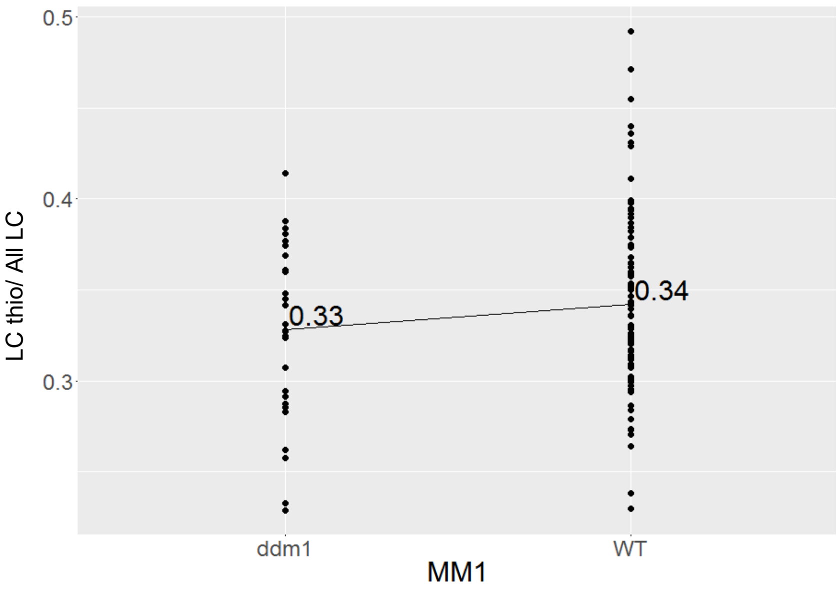

Figure S6: Additive effect of marker one on LC glucosinolates. Y-axis shows the ratio between 7MTH and 8MTO to total LC glucosinolates (7MTH+7MSH+8MTO+8MSO). X-axis shows groups of epiRILs being as *ddm1* mutant in marker one and as WT in marker one. Mean value is written.
