# Supplementary material for: Comparison of the Relative Potential for Epigenetic and Genetic Variation To Contribute to Trait Stability
Source: G3 (Bethesda). 2018 Mar 21;8(5):1733–46. doi: 10.1534/g3.118.200127 (PMC5940164; doi:10.1534/g3.118.200127)
Supplement: Supplementary file 7 [file 1733TableS1.docx]

| **Class** | **Name** | **Abbreviation** |
| --- | --- | --- |
| SC glucosinolates | 3-methylthiopropyl | 3MTP |
| SC glucosinolates | 3-methylsufinylpropyl | 3MSP |
| SC glucosinolates | 4-methylthiobutyl | 4MTB |
| SC glucosinolates | 4-methylsulfinylbutyl | 4MSB |
| SC glucosinolates | 5-methylsulfinylpentyl | 5MSP |
| LC glucosinolates | 7-methylthioheptyl | 7MTH |
| LC glucosinolates | 7-methylsufinylheptyl | 7MSH |
| LC glucosinolates | 8-methylthiooctyl | 8MTO |
| LC glucosinolates | 8-methylsufinyloctyl | 8MSO |
| Indolic glucosinolates | Indolyl-3-methyl | I3M |
| Indolic glucosinolates | N-methoxy-indol-3-methyl | NMOI3M |
| Indolic glucosinolates | 4-methoxy-indolyl-3-methyl | 4MOI3M |

**Table S1: Individual glucosinolates in the three biosynthetic groups SC, LC and Indolic glucosinolates.** The specific glucosinolates measured, their chemical names, biosynthetic group and abbreviation are listed.
